# Episomal virus maintenance enables bacterial population recovery from infection and promotes virus–bacterial coexistence

**DOI:** 10.1093/ismejo/wraf066

**Published:** 2025-04-11

**Authors:** Rodrigo Sanchez-Martinez, Akash Arani, Mart Krupovic, Joshua S Weitz, Fernando Santos, Josefa Antón

**Affiliations:** Department of Physiology, Genetics and Microbiology, University of Alicante, Alicante 03690, Spain; Department of Biology, University of Maryland, College Park, MD 20742, United States; Institut Pasteur, Université Paris Cité, CNRS UMR6047, Archaeal Virology Unit, Paris 75015, France; Department of Biology, University of Maryland, College Park, MD 20742, United States; Department of Physics, University of Maryland, College Park, MD 20742, United States; Institut de Biologie, École Normale Supérieure, Paris 75005, France; Department of Physiology, Genetics and Microbiology, University of Alicante, Alicante 03690, Spain; Institute of Health and Biomedical Research of Alicante (ISABIAL), Alicante 03010, Spain; Department of Physiology, Genetics and Microbiology, University of Alicante, Alicante 03690, Spain; Institute of Health and Biomedical Research of Alicante (ISABIAL), Alicante 03010, Spain; Multidisciplinary Institute of Environmental Studies Ramón Margalef, Alicante 03690, Spain

**Keywords:** pseudolysogeny, Salinibacter, virus–host interactions, modeling, acquired phage resistance

## Abstract

Viruses are ubiquitous in aquatic environments with total densities of virus-like particles often exceeding 10^7^/ml in surface marine oligotrophic waters. Hypersaline environments harbor elevated prokaryotic population densities of 10^8^/ml that coexist with viruses at even higher densities, approaching 10^10^/ml. The presence of high densities of microbial populations and viruses challenge traditional explanations of top–down control exerted by viruses. At close to saturation salinities, prokaryotic populations are dominated by *Archaea* and the bacterial genus *Salinibacter*. In this work we examine the episomal maintenance of a virus within a *Salinibacter ruber* host. We found that infected cultures of *Sal. ruber* M1 developed a population-level resistance and underwent systematic and reproducible recovery post infection that was counter-intuitively dependent on the multiplicity of infection, where higher viral pressures led to better host outcomes. Furthermore, we developed a nonlinear population dynamics model that successfully reproduced the qualitative features of the recovery. Together, experiments and models suggest that episomal virus maintenance and lysis inhibition enable host-virus co-existence at high viral densities. Our results emphasize the ecological importance of exploring a spectrum of viral infection strategies beyond the conventional binary of lysis or lysogeny.

## Introduction

Diverse microorganisms adapted to life at high salt concentrations are found in globally distributed hypersaline environments that comprise ~50% of continental waters [1]. The abundance of prokaryotes increases with salinity, reaching values even higher than 10^8^ cells per milliliter at salinities >25% [2, 3, 4]. The microbial communities in these environments, especially at extreme salinities, are dominated by members of the domain *Archaea* along with *Bacteria* predominantly from the *Salinibacter* genus, with the haloarchaea *Haloquadratum walsbyi* comprising 80%–90%, and *Salinibacter ruber* accounting for 1%–10% of the total cells [5, 6]*. Sal. ruber* was the first bacterium confirmed to grow actively in extreme salinities [7], and global-scale biogeographic studies have revealed that *Sal. ruber* is one of the most prevalent and dispersed bacterial lineages in hypersaline environments. Moreover, it is also characterized by a high intraspecific diversity, which can be caused as a response to an intense viral predation [8, 9, 10]. At saturated salt conditions, hypersaline environments are also characterized by the presence of viral-like particles (VLP) that can reach up to 10^10^ VLP/ml [11], more than three orders of magnitude higher than estimates from surface marine communities [12, 13]. Measured ratios of viruses to microbes increase with salinity (from seawater to highly salt-saturated ponds) with values between 10 and 100 VLPs per cell, and can even reach 300 VLPs per cell [14]. Hypersaline environments at close-to-saturation salinities are typically inhospitable to bacterivorous organisms found in other aquatic habitats, including protists and heterotrophic nanoflagellates [15, 16, 17]. Unusually high viral abundances and virus-to-cell ratios along with reduced bacterivory in hypersaline environments suggest that viral infections and the subsequent modulation of microbial cell fate have a significant role in shaping prokaryotic population dynamics [18].

At the cellular scale, viral infections can lead to a continuum of outcomes spanning pathways that lead to rapid lysis as well as long-term persistence via lysogeny [19]. In the lytic pathway, the virus redirects host metabolism to replicate the viral genome, produce virions, and lyse the host cell, thereby releasing viral progeny to the environment. Many virulent strains of viruses of *Sal. ruber* have been identified [20], suggesting that viral lysis of these cells may be a key, ecological component of high salinity microbial communities. However, the presence of high microbial densities, low bacterivory, and unusually high VLP levels in saturated brines presents a challenge to conventional explanations of top–down control. If extremely high viral densities are achieved through efficient infection and lysis, one might expect strong selection for viral-resistance via kill-the-winner-like mechanisms [21, 22] or even the local collapse of archaeal and bacterial communities (and their viruses) until recolonized via dispersal. Alternatively, prokaryotes and viruses may persist at high levels in hypersaline environments because of sustained production from infected cells or a high stability of non-infectious viruses.

In contrast to rapid lysis, viruses can initiate the lysogenic pathway, integrating their genome into the host chromosome and replicating synchronously with the cell. The provirus (i.e. the integrated viral genome) can reactivate stochastically or in response to changes in host state, re-initiating the lytic pathway leading to the release of virus particles [23, 24]. However, there is increasing recognition of a continuum of lifestyles between the canonical options of lysis or lysogeny [19]. For example, a viral genome can persist as an episome, remaining an independently replicating extrachromosomal unit that coordinates with the host chromosome and equally segregates to daughter cells. Likewise, viruses can initiate persistent infections, including the intermittent release of virions into the environment without cell lysis [25]. Alternatively, genomes of non-temperate viruses can also persist inside host cells without integration as episomes. These non-temperate viruses do not replicate synchronously with their host cell and can be transmitted asymmetrically from mother to daughter cells—this is commonly referred to as pseudolysogeny [26, 27]. Recent sequencing-based studies have identified the ubiquitous presence of functionally virulent phage (typically jumbo phage types) inside bacterial isolates [28]. We interpret these recent findings to suggest that rapid lysis and evolution of resistance as a possible, but not the only, potential explanation for virus–bacterial coexistence at high abundances.

Here, we utilize a *Sal. ruber*-virus model system to explore the mechanistic basis for the persistence and coexistence of high-density populations of viruses and bacterial hosts in hypersaline environments. Initial experiments revealed that virus infections led to a transient bacterial population crash followed by a recovery. However, contrary to our expectations and leveraging a combination of experiments, sequencing, and mathematical modeling, we conclude that bacterial population recovery was not enabled by the emergence of virus-resistant bacterial mutants or lysogeny. Instead, bacterial population recovery was enabled via the initiation of a persistent infection characterized by the episomal maintenance of viruses that protected infected cells from subsequent infection and lysis. As we show, this persistent infection enables lytic viruses to persist at high abundances without necessarily eliminating their hosts, providing new challenges to paradigms of virus–bacterial coexistence.

## Materials and methods

### Growth conditions and nucleic acid extraction

*Sal. ruber* strain M1 was grown aerobically with gentle shaking (60 rpm) at 37°C in 25% SW (sea water) with 0.2% yeast extract, a medium used routinely for halophile growth [29]. *Sal. ruber* M1 was infected with the lytic virus EM1 [20], using a multiplicity of infection (MOI) of 0.01 plaque forming units (PFUs) per cell at time 0 h. Briefly, viruses were mixed with cells at exponential phase (optical density, OD, at 600 nm = 0.3) and incubated during 30 min at room temperature without shaking to facilitate the adsorption. After the adsorption, the abovementioned culture medium was added up to 25 ml and incubated as previously described. Infections were monitored by OD at 600 nm. Non-infected *Sal. ruber* M1 was grown as the control. All experiments were conducted in triplicate. Another curve was made with the same methodology but using a MOI of 0.1 and 10 replicates in both control and infection.

Bacterial pellets of the initial (C_0_, before virus addition) and final cells (C_F_) of the curves performed using a MOI of 0.01 were obtained by centrifugation at 17.000 ×*g* during 10 min. The cells were washed three times with sterile medium, the supernatant was removed, and the cell pellets were stored at −80°C. C_0_ and C_F_ deoxyribonucleic acids (DNAs) were extracted with the DNeasy Blood & Tissue Kit (Qiagen, Hilden, Germany) following the manufacturer’s protocol, and nucleic acids eluted in 70 μl of milli-Q water and quantified using Qubit 2.0 Fluorometer (Life Technologies, Carlsbad, USA).

### DNA sequencing and bioinformatic analysis

DNA was sequenced on a HiSeq System (Illumina) (2 × 150 bp) at Novogene (Novogene, Seur, UK). Primers and adapters were removed from sequences, and reads were filtered based on quality scores using Trimmomatic v0.36.0 [30]. Trimmed reads were assembled using SPAdes v3.13.1 with the trusted option using the reference genome of *Sal. ruber* M1 as scaffold [31]. The mean sequencing depth was calculated by a BLASTn-recruitment analysis where the assembled contigs were used as reference.

For the search for hybrid host-virus reads, a BLASTn of the trimmed reads was performed, using the genome of *Sal. suber* M1 of NCBI as a database (GenBank accession number: NZ_CP030364.1). The reads with >95% ID were filtered by best hit and extracted. No horizontal coverage filter was used. Another BLASTn analysis was performed with these reads using the genome of the EM1 virus of NCBI as a database (GenBank accession number: NC_042348.1) with the same parameters. No hybrid host–virus reads were found.

Mutations generated during the infective process were identified comparing reads from C_F_ with the reference contigs assembled from C_0_ using Geneious software 6.1.8 (Biomatters, Auckland, New Zealand) with a minimum variant frequency of 0.01% and a coverage >25% to the mean coverage.

### PCR and epifluorescence microscopy

Cells from C_0_ and C_F_ were diluted and plated on SW 25% with 0.2% yeast extract agar plates (concentration 2%) and incubated at 37°C for 30–45 days until colonies visualization. Twenty colonies from every point (C_0_ and C_F_) were grown in 2 ml of culture medium at 37°C and 60 rpm. Once the cultures grew to late exponential phase, 200 μl of each were washed three times by centrifugation (see above). DNA was extracted from washed pellets by boiling at 100°C for 10 min after resuspending the cells in 80 μl of milli-Q water. Two parallel polymerase chain reaction (PCR) amplifications were performed with each DNA, using specific primers for *Sal. ruber* M1 and the virus EM1 (Supplementary Table 4). PCR amplifications were carried out in a final volume of 25 μl, containing: 0.75 μl of 1.5 mM MgCl_2_, 2.5 μl of 10X reaction buffer, 0.5 μl of 10 mM dNTPs, 0.1 μl of Taq polymerase (5 U/μl, Invitrogen, Waltham, USA), 0.5 μl of 10 μM primers, 1 μl of extracted DNA, and milli-Q water to complete the final volume. PCR conditions are described in Supplementary Table 5. PCR products were electrophoresed and UV-visualized after staining with ethidium bromide (100 μg/ml).

Three random cultures from C_F_ colonies that were PCR positive for both *Sal*. *ruber* M1 and the EM1 virus (named 1R, 2R, and 3R) were selected for the visualization of extracellular virions by SYBR Gold staining [32]. Briefly, 100 μl of the culture were fixed with formaldehyde (4% final concentration) for 1 h at 4°C. Fixation was stopped by adding phosphate-buffered saline (PBS) 1X up to 1 ml. Twenty microliters (equivalent to 2 μl of sample) were filtered through 0.02 μm Anodisc 25 filters (Whatman Int. Ltd, Maidstone, UK) to retain cells and viruses. Filters were stained with SYBR Gold (25X) for 15 min, washed twice for 1 min with milli-Q water and visualized with an epifluorescence microscope (Leica, type DM4000B).

### Pulsed-field gel electrophoresis and Southern blot

One ml of liquid cultures of C_0_, PCR positive for M1, and C_F_, PCR positive for M1 and EM1, were centrifuged and the cell pellets mixed with agarose at 0.8% (final concentration) to obtain plugs, as detailed in Santos et al., 2007 [33]. Plugs were incubated overnight at 50°C in ESP (0.5 M EDTA, pH 9.0, 1% N-laurylsarcosine, 1 mg/ml proteinase K) for cell lysis and virion disruption and stored at 4°C. DNA was separated by pulsed-field gel electrophoresis (PFGE) on a 1% LE agarose gel in 0.5X TBE buffer, using a Bio-Rad CHEF DR-III System (Bio-Rad, Richmond, USA), under the following conditions: 8–12 s pulse ramp, 6 V/cm, at 14°C for 30 h. After electrophoresis, gels were stained with ethidium bromide (100 μg/ml) for 10 min, washed with distilled water for 30 min, and UV-visualized with a transilluminator.

Agarose gels were transferred to a positively charged nylon membrane (GE Healthcare, Buckinghamshire, UK) as previously described [34]. In parallel, DNA from the EM1 virus was labeled to be hybridized against the membrane using the DIG High Prime DNA Labelling and Detection Starter Kit II (Roche, Mannheim, Germany), following manufacturer’s instructions.

### Growth curves, spot tests, and adsorption assays

*Sal. ruber* M1 wild-type (as a control) and pseudolysogen 1R were grown in liquid, transferred to fresh media (1% v/v) and incubated at 37°C with gentle shaking at 60 rpm. Cellular density was measured by OD at 600 nm. The growth curves of *Sal. ruber* M1 wild-type and pseudolysogen 1R were compared for statistical differences using 99% confidence intervals obtained through the Delta method (Modeling Supplementary Information: Sal. ruber M1 wild-type and 1R (pseudolysogen) growth comparison).

To test the resistance/susceptibility of *Sal. ruber* M1 wild-type and pseudolysogen 1R to the EM1 virus, both hosts were exposed to the virus by a spot test. Four ml of molten 0.7% top agar of 25% SW with 0.2% yeast extract were mixed with 500 μl of bacterial cultures at exponential phase and plated on solid medium. Once solidified, 3 μl of the EM1 virus, tittered at 10^10^ PFUs/ml and diluted 10^2^, 10^4^, and 10^6^ times, were added to the lawn. The spotted plates were left to dry and incubated at 37°C for 10 days. The detection of a clearance zone was interpreted as evidence of viral lysis and the areas exhibiting growth were attributed to resistance against EM1.

An adsorption assay was performed with the EM1 virus and *Sal. ruber* M1 wild-type. Cultures in exponential phase were diluted with fresh medium to an optical density of 0.3 and 2 × 10^8^ cells were mixed with 2 × 10^6^ PFU (MOI = 0.01) or 2 × 10^9^ PFU (MOI = 10) of the EM1 virus. This was considered as the initial time. It was incubated at 37°C and 60 rpm. Aliquots of 150 μl were taken along 2 h (at 15, 30, 45, 60, 90, and 120 min), centrifuged at 17.000 ×*g* for 8 min, and 120 μl of the cells-free supernatant were stored in ice. The number of infective free viruses was measured by plaque assay, diluting the supernatants in sterile SW 25% and mixing 100 μl of each with 500 μl of *Sal. ruber* M1 wild-type in exponential phase. Four ml of top agar were added and this was plated on solid plaques. After solidifying, the plates were incubated at 37°C for 10 days until plaques were visible. The number of free infective viruses (PFU/ml) were counted and represented. A control of the viral decay of the EM1 virus was added, in which the virus was incubated without host. All the experiments were conducted in triplicate.

To get more information about the resistance mechanism, an adsorption assay was performed with the EM1 virus and *Sal. ruber* M1 wild-type and pseudolysogen 1R with a MOI of 0.01 as described above.

To calculate the adsorption rate, the graph of the free infective viruses (PFU/mL) was plotted with respect to time and fit to an exponential function. As the assay was conducted within a 120 min time period and the latent period of the EM1 virus is around 21–22 h, we can assume that there is negligible killing and the susceptible host population stays constant at 2 × 10^8^ cells/ml. We fit the adsorption data to the function and get an adsorption rate 4 × 10^−10^ ml/h (Modeling Supplementary Information: Adsorption rate calculation for M1-EM1 system).

### Infection curves at varying multiplicity of infections and revival of lysis in pseudolysogens

*Sal. ruber* M1 wild-type was grown at 37°C and 60 rpm. The EM1 virus was added at 67 h at middle exponential phase. Cultures were infected with a MOI of 0.01, 0.1, 1, 5, and 10 PFUs per cell in separate experiments. Infections were monitored by OD at 600 nm. *Sal. ruber* M1 was grown without viruses as a control. All experiments were conducted in triplicate.

A total of 2 × 10^8^ cells from the end of each infection curve were transferred to 4 ml of fresh medium (SW 25% with 0.2% yeast extract). This caused all cultures to be diluted to an OD of ~0.1. Cultures were monitored by OD at 600 nm.

### Numerical simulations

All the numerical simulations were performed on Python 3.11.5 through the Jupyter IDE. To solve the model ODEs and obtain the simulated results, we used the numerical integration module *odeint* from the *SciPy* package. To convert the experimental OD measurements to concentrations, we fit a calibration curve using the *curve_fit* module from the *SciPy* package. All the code that can reproduce the simulated results seen in this paper are available on the Github: https://github.com/aranilah/SalruberM1_EM1_recovery/tree/main.

### Transfers of pseudolysogen 1R to fresh media, PCR, and qPCR

In order to assess if the EM1 virus was with generations, pseudolysogen 1R was transferred four times to fresh media. Briefly, pseudolysogen 1R was transferred in triplicates to fresh media (1% v/v) and incubated at 37°C with shaking at 60 rpm, measuring the growth by OD at 600 nm. At the late exponential phase cultures were transferred again to fresh media until a total of 4 transfers. A spot test of each transfer was also carried out as previously mentioned to check if the different transfers of pseudolysogen 1R were still resistant to the virus. Moreover, 100 μl of each transfer were fixed with formaldehyde (0.5% final concentration to avoid PCR inhibition) for 1 h at 4°C. Fixation was stopped by adding PBS 1X up to 1 ml.

To eliminate free viruses, assuming that viruses could have been spontaneously released during the transfer periods, the fixed samples were centrifuged at 17.000 ×*g* during 10 min and washed with PBS five times. PCR reactions for *Sal. ruber* M1 and the EM1 virus were carried out as mentioned above, with 1 μl of the fixed and washed cells as DNA template (first, we checked that the PCR initial thermal shock was sufficient for cell lysis; data not shown).

Once the presence of EM1 was detected in all the transfers, a qPCR was performed to monitor virus concentration along the subsequent transfers. The qPCR assay was conducted using TaqMan hydrolysis probes (Supplementary Table 4) labeled with hexachlorofluorescein (for *Sal. ruber* M1) and fluorescein (for EM1). The experiment was carried out using the standard run in a StepOnePlus PCR System (Life Technologies, Carlsbad, USA) in a 10 μl reaction mixture with PrimeTime Gene Expression Master Mix (Integrated DNA Technologies, Coralville, USA). The reaction contained: 5 μl of 2X Master Mix, 0.2 μl of each 10 μM primer, 0.2 μl of 10 μM TaqMan probe, 1 μl of fixed sample and milli-Q water to complete volume. Conditions are detailed in Supplementary Table 6. The results were analyzed with the Applied Biosystems StepOne Instrument program. All samples were run in triplicate (including the standards and negative controls).

### Plating of pseudolysogen 1R, mutation screening, and spot test

Pseudolysogen 1R at late exponential phase was plated on solid medium (SW 25% with 0.2% yeast extract and 2% agar) to obtain individual colonies. After two months of incubation, 50 colonies developed, which were picked and transferred to 1 ml of liquid media each. The presence of the EM1 virus and the resistance/susceptibility was checked by PCR and spot test as previously described.

To check if the colonies had the mutation affecting the sodium/glucose cotransporter, primers were designed to amplify the specific region (Supplementary Table 7). DNAs of the six selected colonies picked in 1 ml of medium and *Sal. ruber* M1 wild-type (as a control of the mutation) were extracted by boiling as previously described and PCR reactions done as described previously. PCR conditions are described in Supplementary Table 8. PCR products were visualized under ultraviolet light after staining with ethidium bromide (100 μg/ml). Products were sequenced with Sanger method at Stab Vida (Stab Vida, Caparica, Portugal) and analyzed with Geneious software 6.1.8 (Biomatters, Auckland, New Zealand).

Eight strains of *Sal. ruber* (M1, M8, M31, P13, P18, SP38, SP73, and RM158) were exposed to pseudolysogen 1R through spot test as previously described, adding spots of 3 μl of 1R at late exponential phase and diluted 10^2^, 10^4^, and 10^6^ times on the double layer formed by the strains.

### One step growth curve

Burst size was determined after carrying out a one-step growth experiment as detailed in Kropinski, 2018 [35] (Supplementary  Table 9).

## Results

### Systematic and reproducible *recovery of Salinibacter ruber* populations after viral-induced lysis

**Figure 1:**
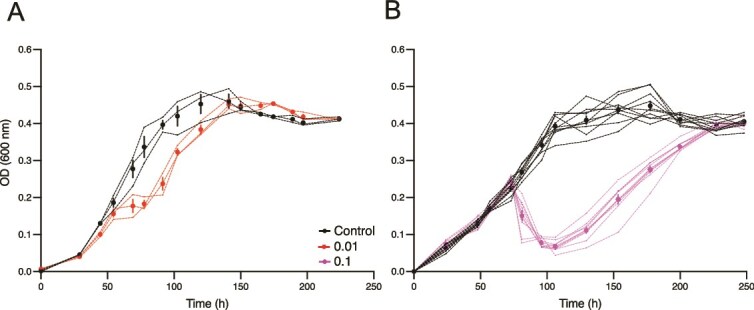
Infection experiments show a reproducible recovery after viral lysis. Infection experiments of *Sal. ruber* strain M1 with the EM1 virus were performed using two different MOIs. Black lines represent uninfected control and red (A, MOI = 0.01) and purple (B, MOI = 0.1) lines represent virus-infected cultures. Each of the individual replicates used are shown as dotted lines. The dots show the mean and the vertical bars the standard error. The experiments were performed with 3 replicates for MOI 0.01 and 10 replicates for MOI 0.1 for both the control and infected cultures. Viruses were mixed with the host at time = 0 h.

We infected *Sal. ruber* strain M1 with the virulent M1EM-1 virus [20] (named henceforth EM1 for convenience) at a MOI = 0.01 in triplicate at time = 0 h (Fig. 1A). In all three replicates, we observed a rapid stabilization in the host optical density (OD) following infection due to lysis, followed by a population-level recovery at 80 h such that infected cultures eventually recovered to the levels comparable to those of the virus-free control, reaching the stationary state ODs (see Methods for further details). This systematic recovery of the population post infection was also observed in other *Sal. ruber*-virus pairs (see Extended Data Fig. 1). This finding was unexpected as the EM1 virus does not encode lysogenic markers (i.e. excisionases and integrases) which would allow its integration into the host chromosome, facilitating superinfection exclusion-mediated population level recovery as seen for some temperate viruses [36]. We further examined this recovery phenomenon by infecting *Sal. ruber* M1 with the EM1 virus at ten-fold higher viral levels (MOI = 0.1) and using more replicates (10 replicates for both control and infection treatment; Fig. 1B). In this case, we observed a sharp decrease in the OD levels of the infected cultures, consistent with the 10x increase in viral pressure (MOI = 0.1 vs. MOI = 0.01). However, we once again observed recovery dynamics comparable in all 10 replicates.

### *Salinibacter ruber* recovery is not consistent with the proliferation of virus-resistant mutants or the integration of viral genomes into bacterial genomes

We hypothesized that the reproducible recovery of infected cell cultures was not due to random mutation of virus-resistance within cells and subsequent proliferation of viral-resistance lineages. This process should lead to large variations (i.e. “jackpots,” *sensu* Luria and Delbrück) in the number of resistant cells [37] and consequent timing of recovery dynamics. Instead, the recovery dynamics were reproducibly consistent. To assess the link between recovery and virus-resistance, all replicates (both the infected cultures and the controls) from the recovered cultures shown in Fig. 1A (named C_F_) were sequenced. The initial *Sal. ruber* M1 used as a host (named C_0_) was also sequenced as a control. We then analyzed and compared the sequenced genomes to evaluate the evidence for selective sweeps of virus-resistance genes in infected replicates.

Only 6 mutations showed a frequency close to 100% (Supplementary Table 1) in all the infected replicates. Of these 6 mutations, 3 involved non-synonymous changes, affecting an ATP-dependent helicase/deoxyribonuclease, an orotate phosphoribosyltransferase, and a sodium/glucose cotransporter. The first two are related to helicase/exonuclease activities and to the synthesis of pyrimidines, respectively, so they did not seem to have a direct relationship with virus resistance. The third mutation, affecting the stop codon in the synthesis of the sodium/glucose cotransporter, could be involved in the resistance, as it affects a transmembrane protein. However, upon further examination, we found that both resistant and sensitive cells had the same mutation, confirming that it was unrelated to resistance. In addition, it was subsequently found that cells that regained susceptibility to the virus retained all the mutations mentioned above (see below and Mutations Supplementary Information for further details). Therefore, no mutations could be unequivocally tied to the observed population recovery phenomenon in the infected cultures.

According to previous studies [38], these dynamics after viral infection could be explained by the integration of the viral genome into the chromosome of its host, yielding lysogens resistant to superinfection with the same virus. Although EM1 lacked any lysogeny-associated traits in its genome, including excisionase and integrases (Supplementary Table 2), the reads of the infected C_F_ cultures were reexamined to search for hybrid host-virus reads, which would signify integration into the chromosome (see Methods). No hybrid reads were found, and therefore recovery cannot be attributed to virus integration. However, we observed a high sequencing depth of the virus genome within infected C_F_ cultures, averaging 146 nt/position, whereas the cellular chromosome exhibited 116 nt/position (Supplementary Table 3). This similar level of sequencing depths suggests that viral genomes are maintained intracellularly in the recovered cultures, albeit not via integration as a prophage.

### Infected cells with episomally maintained viruses comprise part of the recovered *Salinibacter ruber* population

**Figure 2:**
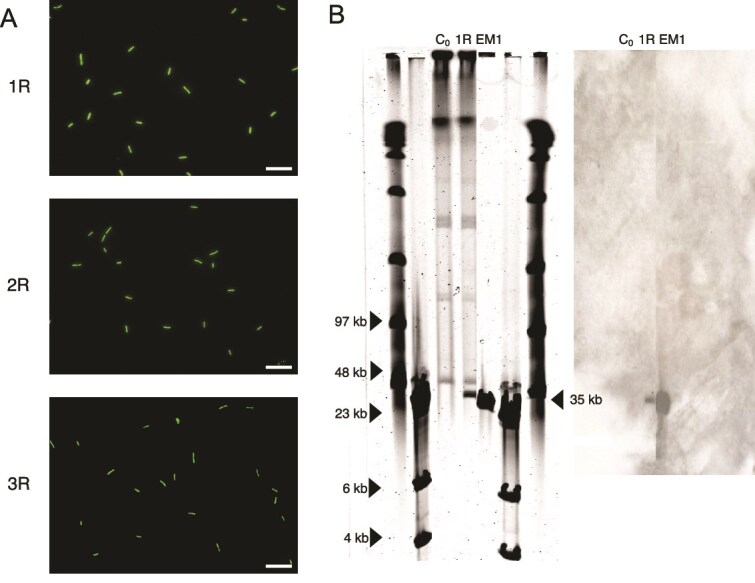
The EM1 virus establishes a pseudolysogenic state in *Sal. ruber* M1. (A) Three randomly selected cultures of the 20 colonies of C_F_, PCR positive for the EM1 virus, that we named 1R, 2R, and 3R, were stained with SYBR gold and observed under epifluorescence microscope to test the presence of extracellular viruses. No extracellular viruses were seen in any of them. Thirty fields were examined per sample. The photos shown here are a representation of each sample. Scale bar: 10 μm. (B) A PFGE was performed (left) comparing initial cells (C_0_) with final colony 1R (lanes 3 and 4 of the gel, respectively). The DNA of the virus was also loaded as a control (lane 5). The PFGE showed a band in 1R with the size of EM1 (35 kb) which was not found in C_0_. A Southern blot (right) with a labeled probe targeting the genome of EM1 confirmed that this band belonged to the virus. Both the PFGE gel and the southern blot images were cropped for visualization purposes, as intermediate lanes contained samples from experiments conducted in the lab unrelated to this work.

The lack of evidence for mutations related to virus resistance or integrated prophage within recovered *Sal. ruber* populations led us to hypothesize that recovered bacterial populations infected by viruses might maintain viral genomes intracellularly in an episomal form. To explore this alternative, we grew colonies from time point C_0_ (initial *Sal. ruber* M1) and C_F_ (final recovered cells). Twenty individual colonies from both C_0_ and C_F_ were grown in liquid medium and PCR-tested for the presence of the EM1 virus. As expected, all C_0_ colonies tested positive with the specific primers for *Sal. ruber* M1 but tested negative for the virus. However, all the final colonies from the infected cultures tested positive for the EM1 virus. Three randomly selected cultures from the C_F_, colonies that tested PCR positive for the virus (which we refer to as 1R, 2R, and 3R, respectively), were stained with SYBR Gold and observed under the epifluorescence microscope (Fig. 2A). After observation of more than thirty fields per culture, extracellular virions were not observed in any case. Hence, although EM1 virus was present in the culture, its virions were not detected extracellularly. The intracellular presence of viral genomes was confirmed by pulsed field gel electrophoresis (PFGE) with DNA from the final colony 1R, but not from the control C_0_ colony (wild-type *Sal. ruber* M1). The PFGE showed a band with the size of the EM1 virus genome (35 kb) in 1R, which was confirmed by Southern blot hybridization with an EM1-specific probe (Fig. 2B). These results, in conjunction with the sequencing data that revealed no signs of integration (no hybrid host-virus reads were found), the lack of integrases and/or recombinases in the genome of EM1 (Supplementary Table 2), supports the hypothesis that the EM1 virus was maintained in an episomal form within *Sal. ruber* M1 recovered populations. To estimate how many cells of the population had the virus, we plated 1R (that we referred as “pseudolysogen”) on solid media and checked how many colonies had the virus (see below).

### Episomal maintenance of viruses protects recovered cells from subsequent infection and viral-induced lysis

**Figure 3:**
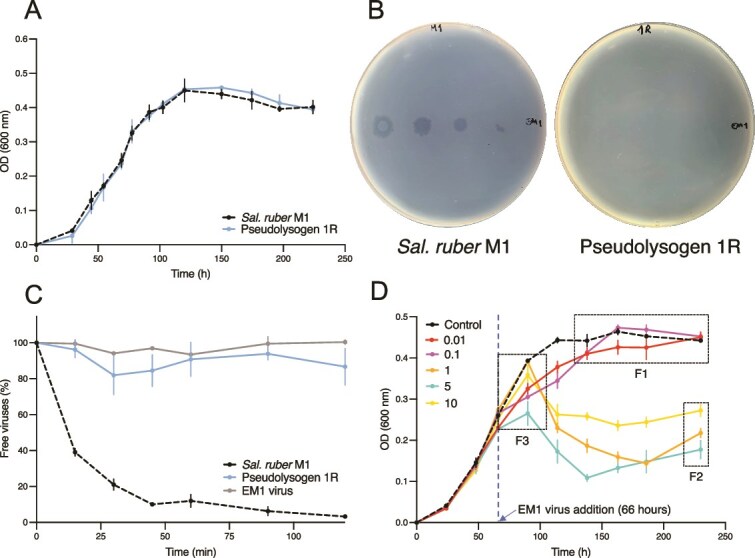
Viral acquisition protects *Sal. ruber* M1 from further infection and has MOI dependent properties. (A) Growth curves (performed in triplicate) of *Sal. ruber* M1 wild-type (black dotted line) and pseudolysogen 1R (blue line) were performed and their growth dynamics were found to have no statistical differences (Modeling supplementary information: Sal. ruber M1 wild-type and 1R (pseudolysogen) growth comparison). (B) Susceptibility to the virus was assessed using a spot test. *Sal. ruber* M1 wild-type (left) and pseudolysogen 1R (right) were exposed to 3 μl of the EM1 virus tittered at 10^10^ PFUs/ml (left part of each plate) and diluted 10^2^, 10^4,^ and 10^6^ (left to right) times. (C) An adsorption assay of the EM1 virus to *Sal. ruber* M1 wild-type (black dotted line) and pseudolysogen 1R (blue line) was carried out with a MOI of 0.01, quantifying the quantity of extracellular viruses through PFU/ml for 120 min. The experiment was also carried out with the virus alone as a decay control (gray line). The experiment was performed by triplicate. Error bars represent the standard error. (D) Infection curves of *Sal. ruber* M1 and the EM1 virus were performed at different MOI. The M1 cultures were infected at 66 h, during the exponential growth phase. The black dotted line represents the non-infected control, and the different colors indicate the MOI, as is indicated in the legend. The experiment results exhibit three key features (see text).

To test whether the acquisition and episomal maintenance of the virus had an influence on the recovered cell growth, we conducted growth experiments on 1R and compared the population dynamics to that of the wild-type *Sal. ruber* M1 (Fig. 3A). We found no statistical differences in the reached OD or growth dynamics between 1R and *Sal. ruber* M1 [Modeling Supplementary Information: Sal. ruber M1 wild-type and 1R (pseudolysogen) growth comparison]. Hence, there is no evidence that viral acquisition provides a direct cost or benefit to host growth.

During typical lysogeny, the prophages can protect hosts from viral infection and lysis via superinfection exclusion [39, 40, 41]. We hypothesize that similar principles may apply to recovered bacterial populations. To test for superinfection exclusion in recovered populations, we conducted spot test assays with 1R and the wild-type *Sal. ruber* M1. In both cases, we plated cells and then overlaid agar plates with spots of EM1 virus serially diluted from 10^10^ PFU/ml to 10^4^ PFU/ml (see Methods). The wild-type strain was susceptible to EM1 infection, whereas 1R exhibited resistance (Fig. 3B). We interpret the results of the spot assay experiment to imply that episomal maintenance of the EM1 virus confers protection against subsequent EM1 infection and lysis. Furthermore, by conducting an adsorption assay with 1R and the wild-type *Sal. ruber* M1, we found that protection against subsequent EM1 infection occurs at the surface level, i.e. preventing effective adsorption (Fig. 3C).

To test how stable is the intracellular maintenance of EM1, we plated the 1R strain and picked colonies at random to test for the presence of the virus by PCR. The results showed that 13 of 17 colonies retained the virus (Mutations Supplementary Information). A spot test with EM1 on the cultures derived from the 17 colonies revealed that only those strains that had lost the virus genome were sensitive to EM1 infection, confirming that the resistance was due to the virus acquisition and episomal maintenance. Subsequent genomic sequencing of 3 of these sensitive and 3 resistant colonies (all derived from 1R) showed that the mutations observed in recovered cultures following the infection (see above) had not been lost and therefore were not involved in the development of resistance to EM1 infection (Mutations Supplementary Information). The reappearance of sensitive strains within the 1R recovered population suggests that the viral genome is not replicating synchronously or faithfully segregated with the host chromosome. Consistently, no marker was found in the virus genome that would indicate cell-synchronous replication and partition, such as the Par system of bacteriophage P1 [42] (Supplementary Table 2). Therefore, we hypothesize the viral persistence inside recovered cells is enabled through asymmetric episomal virus transmission during cell division consistent with a pseudolysogenic state [27]. For the remainder of the manuscript, we refer to bacterial populations post-infection as “recovered” and use the term resistant pseudolysogens to refer to the subset of bacterial populations that have episomally maintained the virus genome and are resistant to subsequent infection and lysis by the same virus.

### Bacterial population recovery depends non-monotonically on multiplicity of infection

The spot assay experiment yielded a counter-intuitive result. When high virus concentrations were added on the plates of wild-type *Sal. ruber* M1 at levels between MOI of 10–100, we observed growth of cells in the center of spots. This suggests that there may be a MOI dependence to the initiation of pseudolysogeny, akin to that found in classic studies of lysogeny in which the probability of lysogeny increases with increasing MOI [24, 43, 44]. Hence, cultures with cells infected by multiple viruses may be more likely to yield pseudolysogens which can grow even in the presence of viruses. To explore the MOI-dependence of recovery further, *Sal. ruber* M1 was infected with EM1 at MOIs ranging from 0.01 to 10 viruses per host cell. The M1 population was infected during its exponential growth phase (66 h). The population dynamics (Fig. 3D) exhibit dip and recovery dynamics for MOI 1, 5, and 10 in a similar fashion to the initial infection curve experiment. In contrast, population dynamics for MOI 0.01 and 0.1 solely exhibit growth, albeit slightly slower than in the control population. The final cell densities in the experiments with MOI 1, 5, and 10 were non-monotonically related to MOI, i.e. the OD values were 0.22 +/− 0.01, 0.18 +/− 0.02, and 0.27 +/− 0.01 for MOI 1, 5, and 10, respectively. We expected that higher MOI should increase virus encounters with host cell and lead to higher levels of cell mortality. Therefore, we hypothesize that the final host concentration when infected at MOIs of 1 and 5 is greater or equal to final host concentrations at a MOI of 10. Using a one-sided *t*-test with a Holm–Bonferroni correction for multiple comparisons, we reject the null hypothesis (*P*-value = 0.01 for MOI 10 vs MOI 5, *P-*value = 0.02 for MOI 10 vs 1). We interpret this to mean that the final host concentration at MOI 10 is greater than the final host concentrations at MOI 1 and 5. We could not reject the null hypothesis that the OD values at time 230 h for MOI 1 vs 5 were statistically different (two-sided *t*-test, *P*-value = 0.206). These results raise the question: if EM1 generates resistant pseudolysogens [as was confirmed in the spot assay experiment for 1R (see Fig. 3B)], then why would recovery dynamics depend non-monotonically on MOI?

The onset of superinfection exclusion may be rapid if extracellular virion binding interferes with subsequent viral attachment, but often takes time to develop post-infection, e.g. due to lipoproteins binding to receptor sites to block further viral attachment at the surface [39, 40]. Therefore, we hypothesize that multiple EM1 viruses can jointly infect the same *Sal. ruber* cell, provided the infections occur before superinfection exclusion develops. For example, given a population-level MOI of 10 and assuming that all virions have adsorbed to the cell and injected their genomes, infection would yield an average of 10 viruses per cell with a standard deviation of ~3. To test whether superinfection was possible in this system, we performed adsorption assays with the wild-type *Sal. ruber* M1 and EM1 at MOI of 0.01 and 10 (Extended Data Fig. 2A). In the case of MOI 0.01, the decrease of free viruses was equivalent to that expected in theory for complete adsorption of virions onto cells (i.e. ~1% of cells should be infected). In the case of MOI 10, the exponential decrease of free viruses plateaus at ~20%, implying that 80% of virions adsorbed to cells, consistent with ubiquitous superinfection (i.e. the average cellular-level MOI would be 8). The fact that 100% of the virions do not adsorb at MOI 10 implies there are limits to viral superinfection. The reasoning behind the limit to the number of viruses per cell could be due to competition for receptor sites, or perhaps explicit spatial limitations. We conclude that superinfection of *Sal. ruber* M1 is possible by the EM1 virus given sufficiently short intervals between infection events, even if the development of a superinfection exclusion state prevents subsequent infection over the long term in resistant cells with episomally maintained viruses.

### Scaling-up the dynamical effects of episomal maintenance of viruses from cells to populations

**Figure 4:**
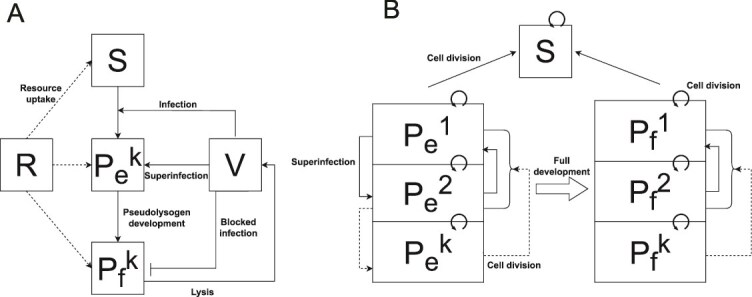
Schematic of nonlinear dynamics population model. (A) Schematic representation of the ODE model, illustrating the resource level and denotes the free virus concentration. The host population is split into three classes: sensitive, early pseudolysogen, and fully developed pseudolysogen, where *k* is the number of viral genome copies within the host cell. (B) Dynamics of pseudolysogen growth and superinfection assuming primary infection has already occurred (primary infection: *S → P_e_^1^*). Early pseudolysogens get superinfected causing → transitions, whereas fully developed pseudolysogens are resistant to superinfection at the surface level (denoted by “blocked infection” arrow). Pseudolysogen cell division for both early and fully developed pseudolysogens randomly splits the viral genome of the parent, causing → transitions where *k’* is a randomly chosen positive integer that is *≤ k* (*P^0^* is virus-free and is therefore equivalent to *S*). Early stage pseudolysogens eventually transition into fully developed pseudolysogens that still cell divide but are resistant to superinfection.

The contextual impact of superinfection raises questions on how pseudolysogeny modulates recovery dynamics, in light of three key empirical observations (Fig. 3D). First, the *Sal. ruber* M1 populations infected at MOI 0.1 and 0.01 do not exhibit an observable population decline. Second, the M1 host population recovers to a higher population density when infected at MOI 10 compared to MOI 5 and MOI 1, highlighting a non-monotonic relationship between viral levels and population-level lysis. Third, when infected at MOI 10, the *Sal. ruber* M1 population does not crash immediately upon infection and maintains persistent transient growth post infection. To connect mechanisms occurring at cellular-scales with these population-level dynamics, we developed a nonlinear ordinary differential equation (ODE) model of the EM1 virus interacting with *Sal. ruber* M1 hosts (Fig. 4). The model includes dynamics of resources (*R*), sensitive cells (*S*), pseudolysogens (*P*), and virus particles (*V*) [45]. We separate pseudolysogens into “early” (*P_e_*) and “fully developed” pseudolysogens (*P_f_*), where viruses can infect sensitive cells and early pseudolysogens (consistent with findings of Extended Data Fig. 2A) which then transition to become resistant, *P_f_* types (consistent with findings of Fig. 3B). We account for cellular-level multiplicity of infection of the pseudolysogens through a superscript ^[*k*]^ where *k* denotes the number of viral genomes per cell e.g. *P_e_* [2] denotes the number of early pseudolysogens with 2 viral genome copies. The number of viruses able to infect a single cell is limited, consistent with adsorption results from Extended Data Fig. 2A (details in Extended Data Fig. 2B). Cell division for both sensitive cells and pseudolysogens is modeled as a function of resource consumption. Full details of the model specification and parameters are provided in the Supplement (Modeling Supplementary Information: Main model).

**Figure 5:**
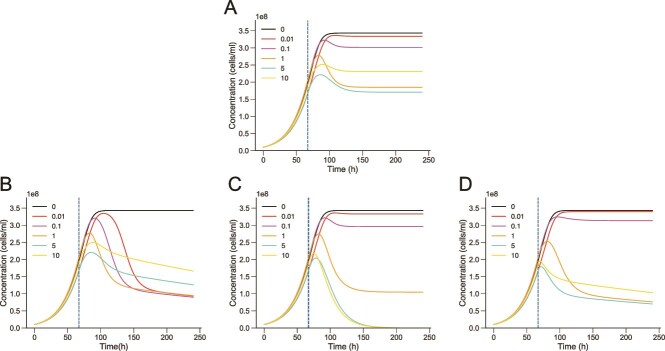
Simulated population dynamics recapitulate experimental features through implementation of lysis inhibition and pseudolysogenic cell division. (A) Simulated host population dynamics (using the ODE model proposed (Modeling supplementary information: Main model) to successfully recapitulate the experimental features highlighted in Fig. 3D. Subfigures B, C, and D graphically show how decoupling features from the model prevents simulations from capturing experimental features. (B) Simulated host population dynamics with lysis rates independent of resource levels. (C) Simulated host population dynamics with lysis rates independent of cellular MOI. (D) Simulated host population dynamics without pseudolysogen cell division.

Simulated population dynamics (Fig. 5A) recapitulate the key empirical observations (Fig. 3D). The match between theory and experiment is driven by the following mechanisms. First, the model assumes that host lysis decreases with decreasing resources [46, 47]. We show that decoupling host lysis from resource concentrations leads to a rapid crash in *Sal. ruber* dynamics among the low MOI populations that is inconsistent with experimental observations (Fig. 5B). Second, the model assumes that cellular lysis rates decrease as a function of increasing cellular MOI [48, 49]. We show that if lysis rates are independent of cellular MOI, then population crashes are most apparent at the highest MOI and decrease with decreasing MOI, inconsistent with experimental observations (Fig. 5C). Third, we assume that pseudolysogens divide and that their daughter cells can inherit viral genomes, albeit randomly, from the parent cell (akin to random segregation of episomal elements [50]). We show that if pseudolysogens do not divide then the *Sal. ruber* population at MOI 10 (yellow curve) immediately dips upon infection at 66 h (blue dotted line) and there is no persistent transient growth, thereby inconsistent with the experimental dynamics (Fig. 5D). In Modeling Supplementary Information: Subsets of main model with corresponding dynamics, we explore all potential combinations of the presence/absence of each mechanism and find that the model only recapitulates observed dynamics when all three mechanisms operate jointly. Hence, we contend that each of these elements (resource-dependent lysis, latent period delays in multiply infected cells, and pseudolysogen cellular division) are necessary to explain experimentally observed dynamics of the EM1 virus and its host across three orders of magnitude difference in initial ratios of virus to host. The details of how all the model features are implemented is highlighted under Modeling Supplementary Information: Key model features and Modeling Supplementary Information: Viral passage in superinfected pseudolysogens. We observe the results are robust to variation in the variability associated with the passage of episomally maintained viruses to daughter cells during replication (Modeling Supplementary Information: Episomal viral replication).

### Episomal maintenance of viruses and the population-scale maintenance of virus–bacterial coexistence

We set out to explore the extent to which episomal maintenance of viruses inside recovered bacterial populations could enable virus–bacterial coexistence over longer time scales. The persistence of the virus within the pseudolysogen population was tested by transferring pseudolysogen 1R every 9–10 generations to fresh medium. We made four such transfers, resulting in ~40 generations of pseudolysogen growth and cell division (Extended Data Fig. 3A). At the end of the exponential phase of each transfer, we conducted a PCR test and found that the virus was not lost over time (Extended Data Fig. 3B). Upon qPCR analysis, we found that the ratio of virus genome/chromosome was close to 1 in the first transfer and upon further transfers, the ratio increased to 2–3 virus genomes per chromosome of M1 and was maintained at this level (Extended Data Fig. 3C). To determine whether viral resistance was maintained within the population, cells from each of the aforementioned transfers were exposed to EM1 through a spot test. We found that the cells from every transfer exhibited resistance to additional infection (Extended Data Fig. 4). Next, to observe how the pseudolysogens might ecologically interact with other *Sal. ruber* strains, we conducted spot tests in which eight strains of *Sal. ruber* used routinely in our laboratory were individually exposed to pseudolysogen 1R. We observed that *Sal. ruber* M1, the only strain sensitive to the EM1 virus, was killed whereas the rest of the seven strains were maintained (Extended Data Fig. 5). Similar dynamics have been observed with temperate viruses, where lysogens induce and release free virions as a weapon to kill sensitive cells in shared environments [51]. We hypothesize that a subset of pseudolysogens of *Sal. ruber* reactivate the lytic pathway, releasing virions that infect sensitive competitors, generating a mix of pseudolysogens and increasing the level of extracellular virions.

## Discussion

Here we explored the dynamics arising from interactions between a virulent virus (EM1) and one strain of a prevalent and globally dispersed bacterial host found in hypersaline environments (*Sal. ruber* M1). We found that *Sal. ruber* M1 populations reproducibly recover from infections by EM1 viruses across three orders of magnitude variation in MOI. M1 bacterial populations recover to higher densities when infected with more viruses (MOI = 10) than with less viruses (MOI 1 or 5), recovering to densities comparable to those of non-infected controls for MOI levels of 0.1 or 0.01. Both sequence and experimental evidence suggest that recovered populations have a significant fraction of cells with episomally maintained virus genomes that pass asymmetrically from mother to daughter cells in the absence of synchronized replication with their cellular hosts. Subsequent infection experiments revealed that these cells with episomally maintained virus genomes (i.e. pseudolysogens) are resistant to infection and lysis by the original virus, providing an opportunity to explore mechanisms by which infection recovery could stabilize bacteria-virus feedback. We developed and analyzed a nonlinear population dynamics model that recapitulated key features of the MOI dependent recovery dynamics that depend on the potential joint infections of cells, lysis inhibition via superinfection immunity, and lysis inhibition due to resource limitation. Altogether, the initiation and maintenance of pseudolysogeny stabilizes short-term population dynamics, providing a mechanism for long-term coexistence of viruses and their bacterial hosts in extreme environments.

The current study connects cellular-level interactions to feedback mechanisms that stabilize virus-host population dynamics that could otherwise lead to population collapse, especially at high MOI values. Previous studies have shown similar recovery dynamics in different virus-host systems [52, 53, 54, 55], but there has been limited effort to explain the mechanisms responsible. Studies observing recovery dynamics phenomenon with temperate viruses have pointed to emergence of resistant lysogens which are protected against further infections [36, 38]. However, bacterial population recovery has also been observed in viruses without any lysogeny markers as well [56, 57], in which the emergence of resistance has been overlooked or simply attributed to spontaneous mutations of the host [58, 59]. Here, recovery dynamics could not be attributed to lysogeny given the use of the *Sal. ruber* M1-EM1 system where EM1 is virulent. The recovery dynamics observed here was also highly reproducible, inconsistent with recovery due to the emergence and expansion of spontaneous resistant mutants that grow from low to high frequency where substantial variability is a hallmark feature of dynamics [37]. Furthermore, our analysis revealed no clear resistance-associated mutations in the recovered M1 populations. Similarly, we also identified lysis inhibition at high MOI and/or when resources are limited as an essential part of explaining the MOI-dependence of recovery dynamics. Lysis inhibition has commonly been noted in infection of *Escherichia coli* by T4 [48, 49, 59, 60], with some studies suggesting that it plays a key role in the maintenance and development of *E. coli*-T4 pseudolysogens [27, 60]. We hypothesize that lysis inhibition could similarly be a key feature of pseudolysogeny in the *Sal. ruber* M1-EM1 system, allowing for persistent infection in starved conditions and/or under high MOI.

Our combined study comes with caveats. We focused primarily on contextual benefits of pseudolysogeny, noting that identifying direct impacts of episomal maintenance of viruses on cellular fitness has limited support [61]. Although we found that there were no statistical differences between the growth rate of pseudolysogens and wild-type host cells, we found that pseudolysogeny provided surface-level protection against further infection. Furthermore, we found that loss of episomally maintained viruses renders pseudolysogens sensitive to further infection. Hence, viral acquisition comes with a cost (the risk of lytic infection) but is key to enabling surface-level resistance and population level recovery. More work is needed to understand when pseudolysogens become resistant and the cellular mechanisms underlying transient protection to infection. We also found that *Sal. ruber* strains that were sensitive to the EM1 virus were killed in the presence of pseudolysogens. Similar dynamics have been observed with temperate viruses of *Roseobacter*, where lysogens induce and release virions to kill competing sensitive strains in head-to-head experiments [51]. We hypothesize that *Sal. ruber* M1 pseudolysogens may switch from a maintenance to an actively infected state, e.g. when episomal numbers are low and/or when resources become available. Again, future work will be needed to elucidate the cellular drivers of lytic initiation and the extent to which intrinsic or extrinsic heterogeneity plays a role in the lysis of pseudolysogens that release EM1 viruses that may then infect and kill competing sensitive strains and potentially initiate new pseudolysogens.

This study reveals how feedback mechanisms at the cellular scale can transform population dynamics leading to non-monotonic relationships between virus infection pressure and bacterial impacts. Put simply: for strictly lytic viruses, more infection does not always result in more killing. Within the *Sal. ruber* M1-EM1 bacteria-virus model system, we have identified feedback mechanisms that limit lysis, whether because of multiple infections, resource limitation, or the establishment of an episomally maintained virus (i.e. a pseudolysogen). Each of these mechanisms stabilizes virus-bacterial population dynamics. We recognize that it is challenging to move from experimental settings to extreme field conditions, hence we are forced - for now - to speculate that pseudolysogeny may be an evolutionarily adaptive strategy for obligately lytic viruses to preserve hosts until environmental conditions improve or host density increases [26, 27, 62]. Depending on the durability of viruses extracellularly, such a balance between infection and production may help to explain virus:host ratios ranging from 10 to as high as 300 in the face of fluctuating conditions [14]. More broadly, it may be of some value to explore other, related cellular mechanisms that stabilize virus-host population dynamics, enabling reproducible recovery dynamics seen in ecosystems outside hypersaline environments, such as the bloom-bust dynamics observed in oceanic *Emiliania huxleyi* populations after infection by EHV at high MOI [52, 63] or the long-term persistence of crAss-like phage crAss001 and its host [64]. Overall, the results from this work highlight the ecological relevance of exploring a continuum of viral infection strategies beyond the dichotomous options of lysis or lysogeny.

## Supplementary Material

Extended_Data_Fig_1_wraf066

Extended_Data_Fig_2_wraf066

Extended_Data_Fig_3_wraf066

Extended_Data_Fig_4_wraf066

Extended_Data_Fig_5_wraf066

ISMEJ-D-24-01407_Supplementary_tables_wraf066

ISMEJ-D-24-01407_Modeling_Supplementary_Information_wraf066

ISMEJ-D-24-01407_Mutations_Supplementary_Information_wraf066

## Data Availability

The raw files used for mutation analysis were deposited in the NCBI database with BioProject accession ID PRJNA1136859. Raw sequences of the Sanger sequencing of the mutation affecting the sodium/glucose cotransporter can be found in the *Mutations Supplementary Information.*

## Code availability

The code used in the study is available via GitHub at: https://github.com/aranilah/SalruberM1_EM1_recovery/tree/main
