# Supplementary figures and images for: Episomal virus maintenance enables bacterial population recovery from infection and promotes virus–bacterial coexistence
Source: ISME J. 2025 Apr 11;19(1):wraf066. doi: 10.1093/ismejo/wraf066 (PMC12064560; doi:10.1093/ismejo/wraf066)

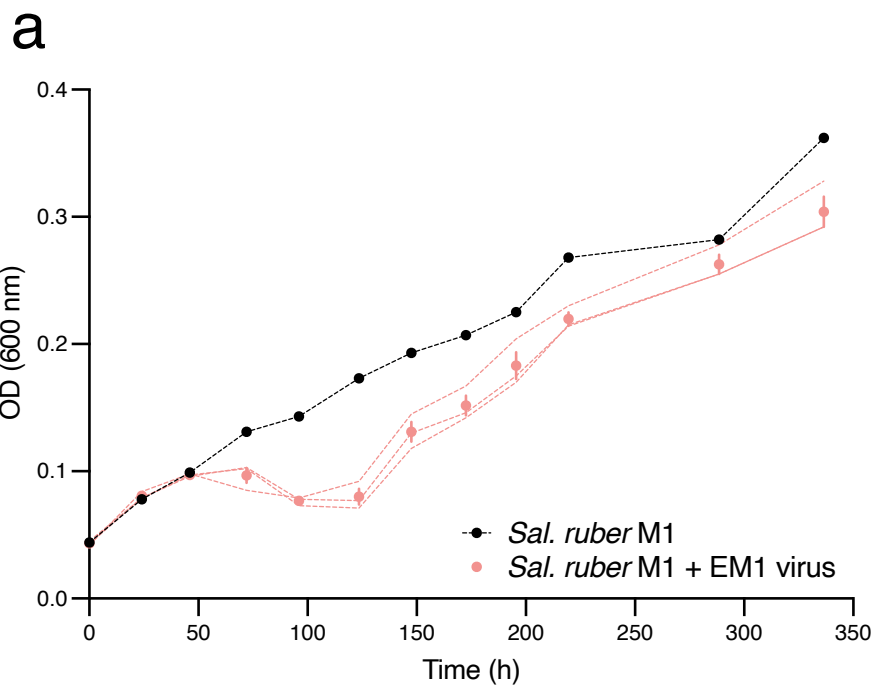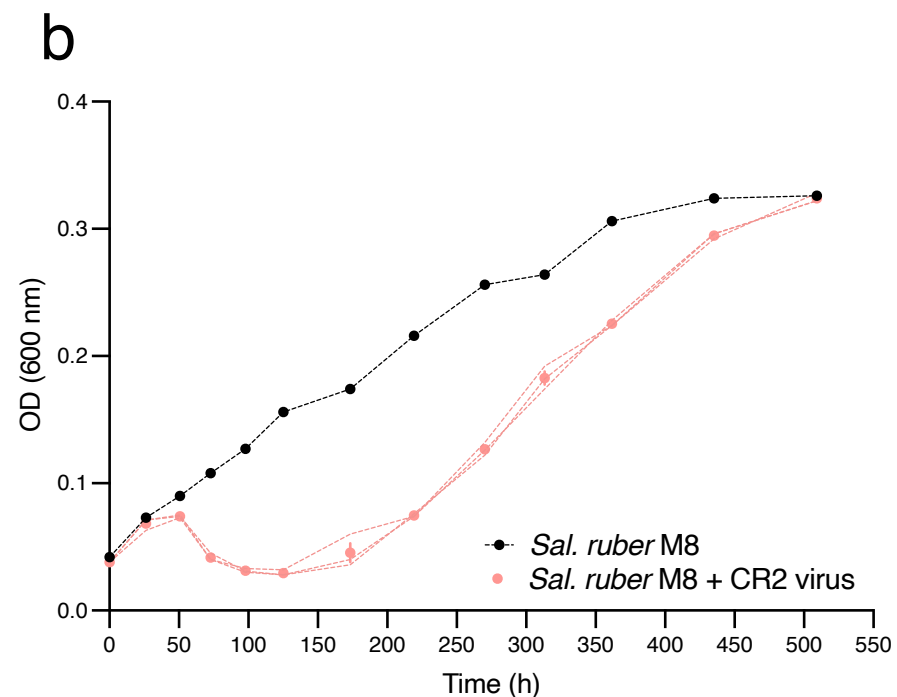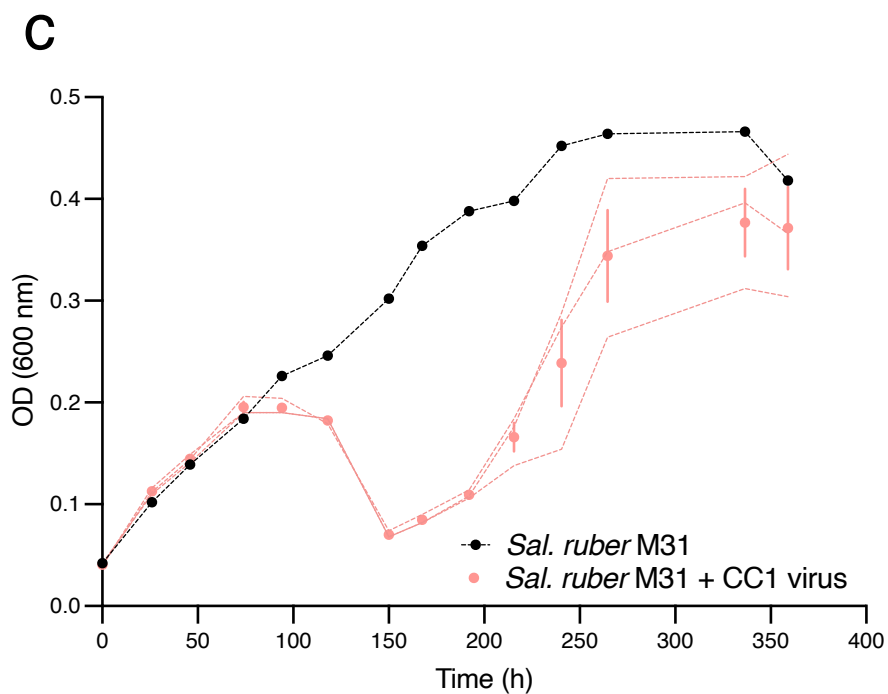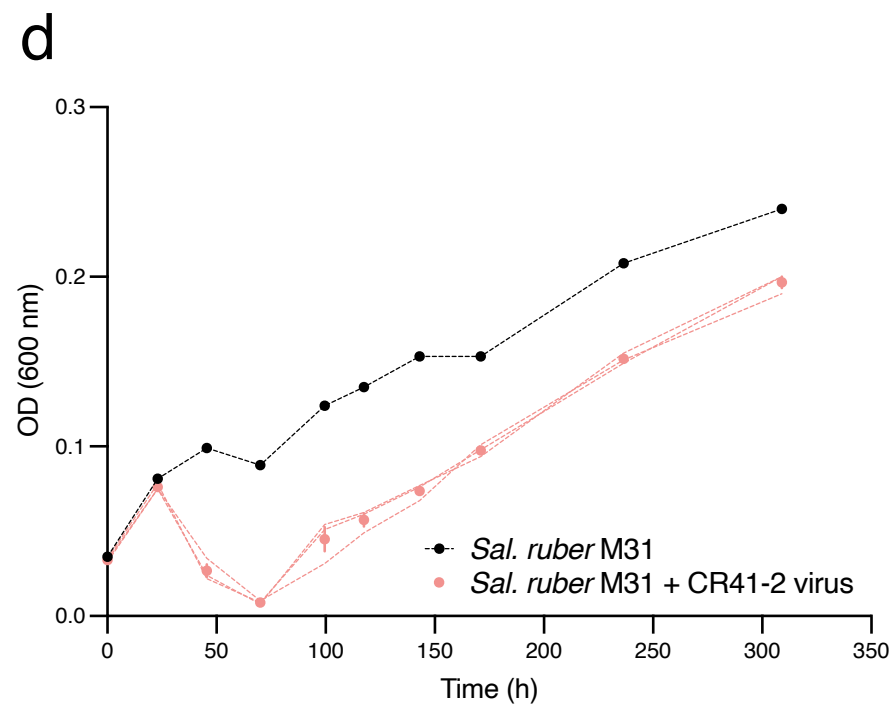

Supplement: Extended_Data_Fig_1_wraf066 [file extended_data_fig_1_wraf066.pdf]

**a**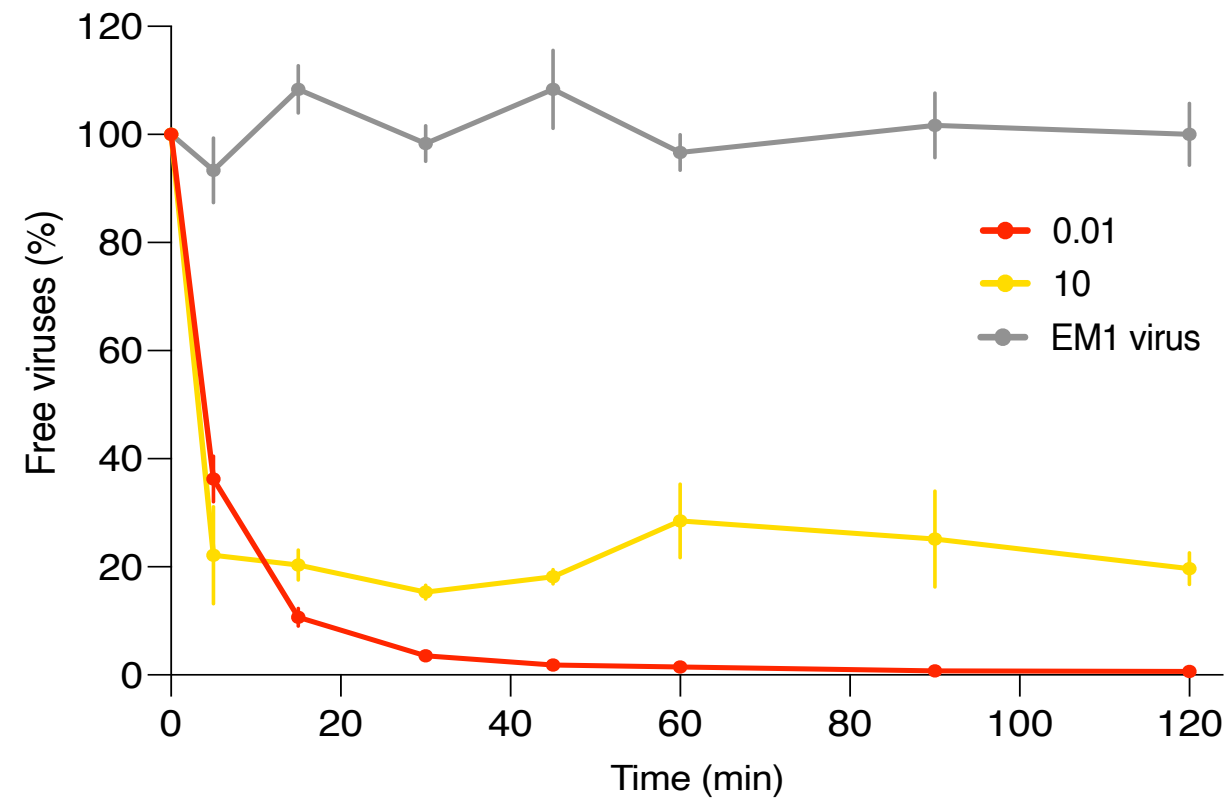**b**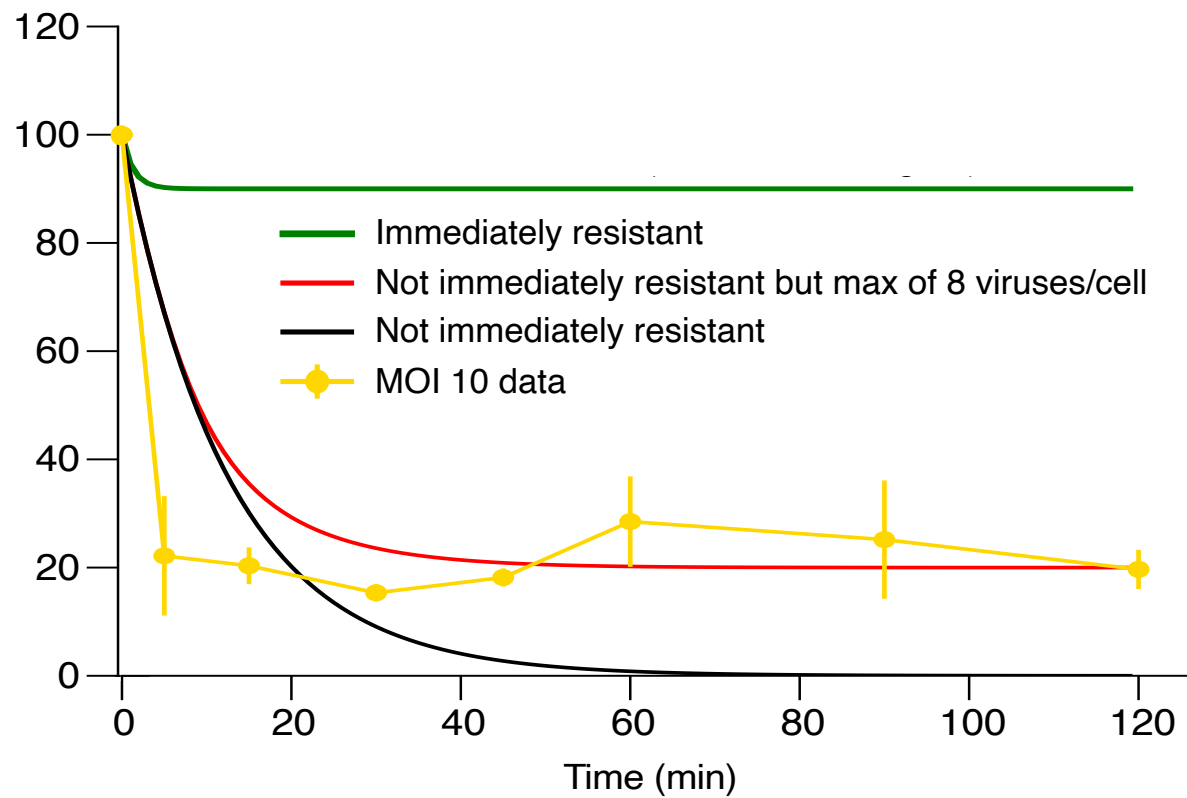

Supplement: Extended_Data_Fig_2_wraf066 [file extended_data_fig_2_wraf066.pdf]

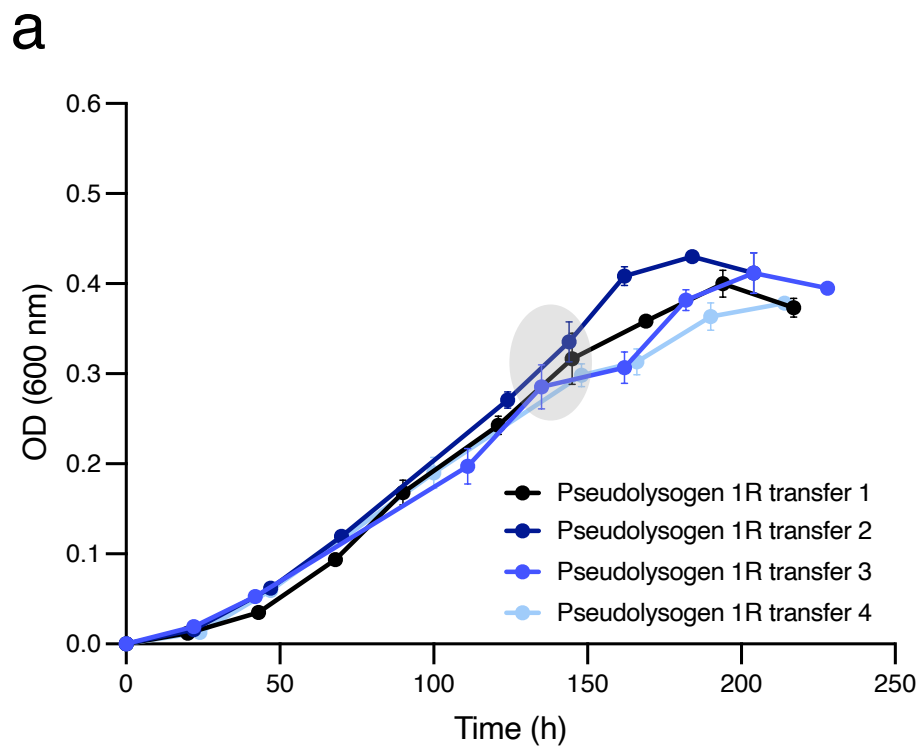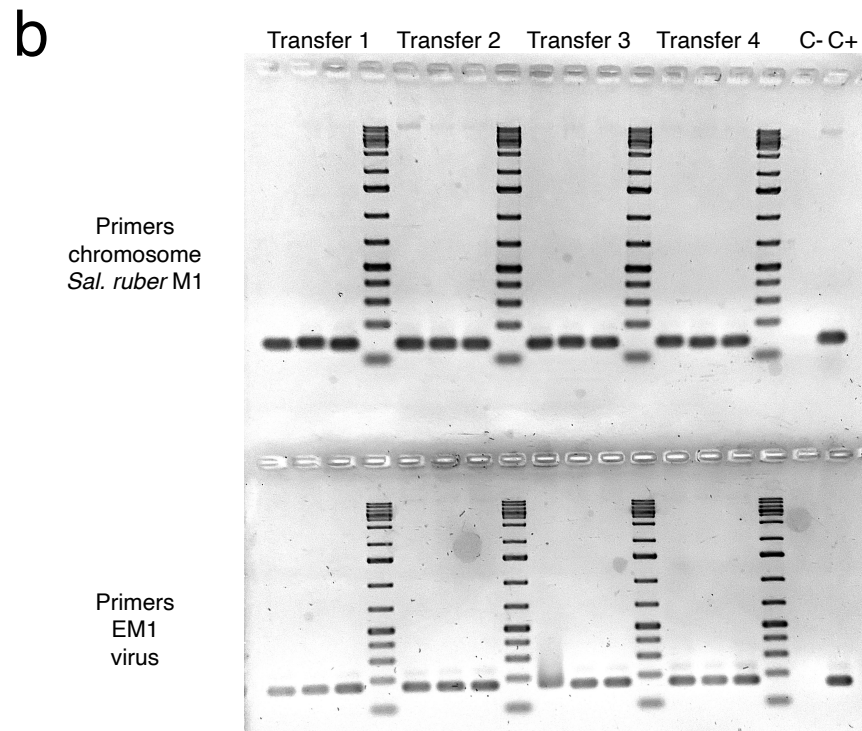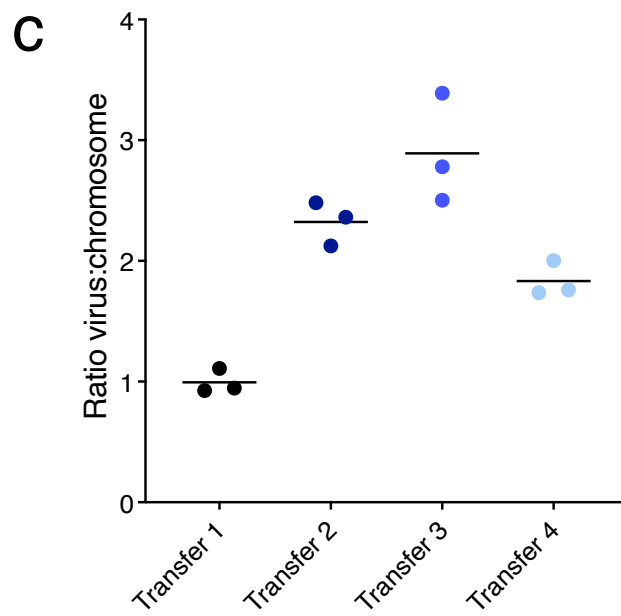

Supplement: Extended_Data_Fig_3_wraf066 [file extended_data_fig_3_wraf066.pdf]

Transfer 1

Transfer 2

Transfer 3

Transfer 4

Control

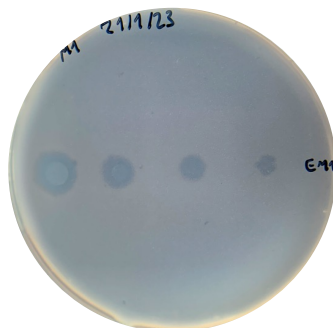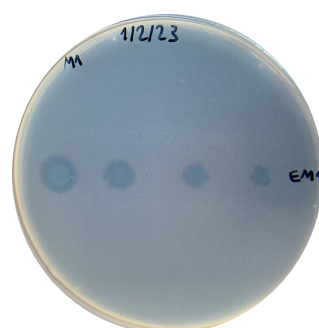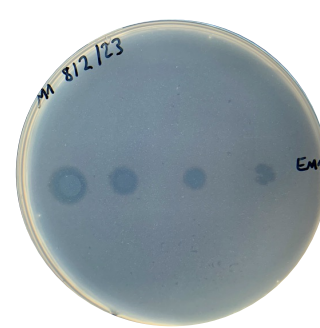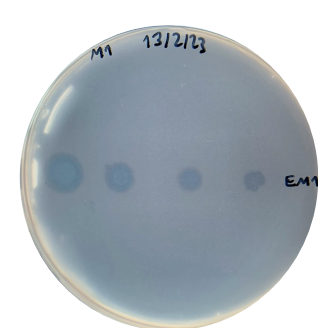

Replicate 1

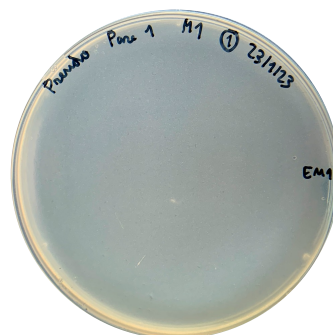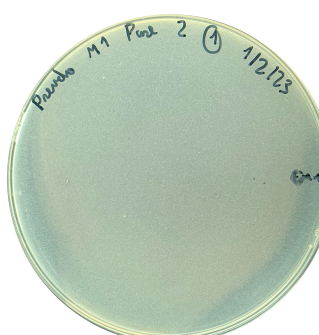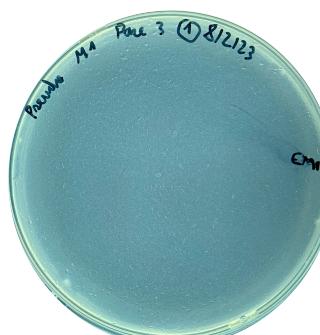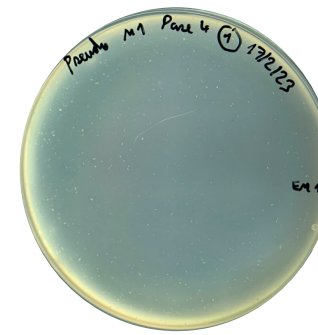

Replicate 2

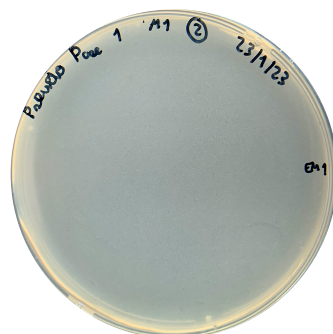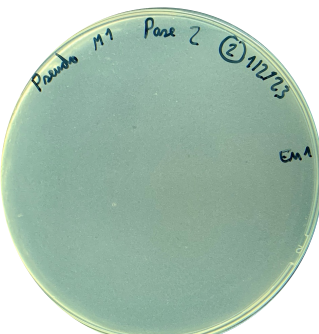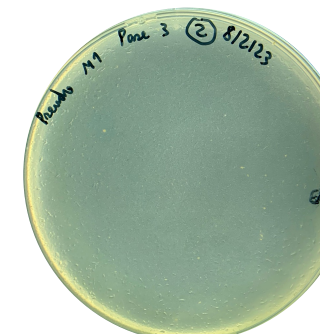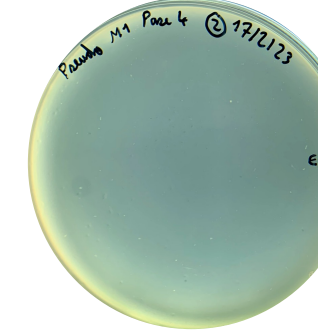

Replicate 3

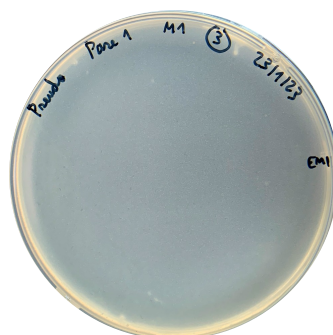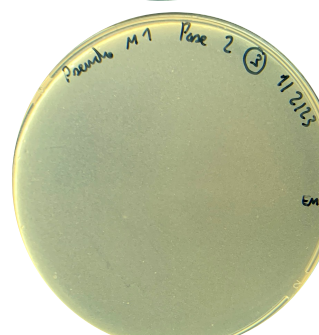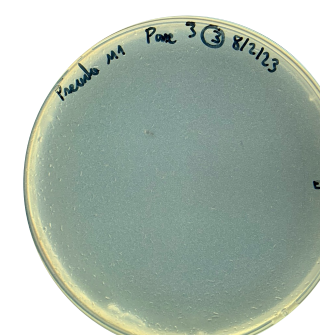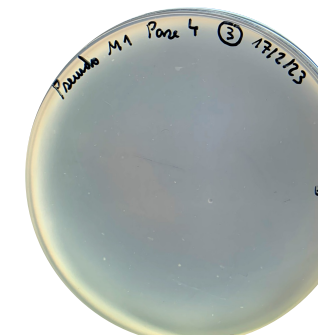

Supplement: Extended_Data_Fig_4_wraf066 [file extended_data_fig_4_wraf066.pdf]
