# Supplementary material for: Episomal virus maintenance enables bacterial population recovery from infection and promotes virus–bacterial coexistence
Source: ISME J. 2025 Apr 11;19(1):wraf066. doi: 10.1093/ismejo/wraf066 (PMC12064560; doi:10.1093/ismejo/wraf066)
Supplement: Extended_Data_Fig_5_wraf066 [file extended_data_fig_5_wraf066.pdf]

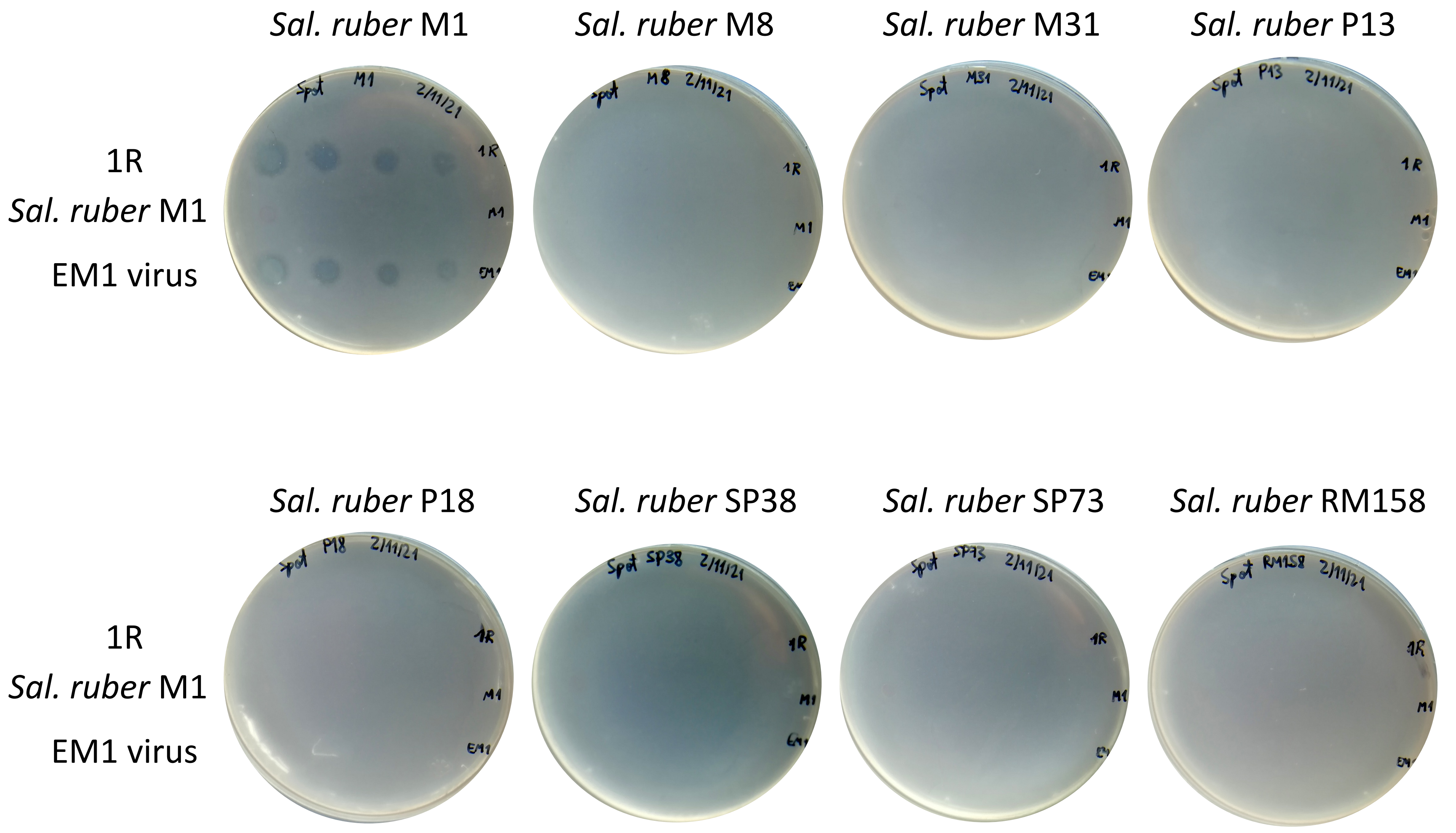
Sal. ruber M1
Sal. ruber M8
Sal. ruber M31
Sal. ruber P13
1R
Sal. ruber M1
EM1 virus
Sal. ruber P18
Sal. ruber SP38
Sal. ruber SP73
Sal. ruber RM158
1R
Sal. ruber M1
EM1 virus
