# Supplementary material for: Episomal virus maintenance enables bacterial population recovery from infection and promotes virus–bacterial coexistence
Source: ISME J. 2025 Apr 11;19(1):wraf066. doi: 10.1093/ismejo/wraf066 (PMC12064560; doi:10.1093/ismejo/wraf066)
Supplement: ISMEJ-D-24-01407_Supplementary_tables_wraf066 [file ismej-d-24-01407_supplementary_tables_wraf066.pdf]

Supplementary Tables: Episomal virus maintenance enables bacterial population recovery from infection and promotes virus-bacterial coexistence

Supplementary Table 1

Product	Position	Protein Effect
ATP-dependent helicase/deoxyribonuclease subunitB	56473	Substitution
Hypothetical protein	858339	None
Sodium/glucose cotransporter	1784378	Extension
Non-codificant region	227,834	None
Orotate phosphoribosyltransferase	2371568	Substitution
Hypoxanthine-guanine phosphoribosyltransferase	3465140	None

Supplementary Table 2

gene_ID	gene_start	gene_end	gene_length	Protein prediction
gene_1	1	237	237	Hypothetical protein
gene_2	729	914	186	DNA-binding HTH domain-containing protein
gene_3	911	2371	1461	Hypothetical protein
gene_4	2397	3746	1350	Structural protein
gene_5	3829	5040	1212	Hypothetical protein
gene_6	5045	6187	1143	Putative head morphogenesis protein
gene_7	6396	7133	738	Head scaffolding protein
gene_8	7209	8033	825	Major head protein
gene_9	8061	8498	438	Hypothetical protein
gene_10	8502	8831	330	Minor capsid protein
gene_11	8835	9149	315	Neck protein Ne1
gene_12	9151	9579	429	Phage minor tail protein
gene_13	9579	10418	840	Structural protein
gene_14	10498	10851	354	Hypothetical protein
gene_15	10941	11210	270	Hypothetical protein
gene_16	11200	13431	2232	Minor tail protein
gene_17	13450	18009	4560	Long tail fiber proximal subunit
gene_18	18299	18661	363	Peptidase
gene_19	18658	19095	438	Hypothetical protein
gene_20	19076	19663	588	Hypothetical protein
gene_21	19677	20018	342	Transcriptional repressor
gene_22	20679	21395	717	DNA-binding HTH domain-containing protein
gene_23	21482	21718	237	Hypothetical protein
gene_24	21822	22013	192	Hypothetical protein
gene_25	22267	22530	264	Hypothetical protein
gene_26	22542	23498	957	Nuclease domain-containing protein
gene_27	23905	24336	432	Structural protein
gene_28	24409	24861	453	Primosome PriB/single-strand DNA-binding
gene_29	24893	25141	249	Single stranded DNA-binding protein
gene_30	25316	25678	363	DNA-binding HTH domain-containing protein
gene_31	25828	26331	504	Putative nuclease
gene_32	26495	26761	267	Hypothetical protein
gene_33	26758	26970	213	Hypothetical protein
gene_34	26967	27326	360	RusA-like Holliday junction resolvase
gene_35	27337	28434	1098	DNA polymerase III beta subunit
gene_36	28494	30764	2271	Topoisomerase-primase domain-containing protein
gene_37	30955	31755	801	GIY-YIG nuclease family protein
gene_38	31978	33180	1203	DNA modification methylase
gene_39	33534	34505	972	Deoxynucleoside monophosphate kinase
gene_40	34600	34959	360	Hypothetical protein
gene_41	34968	35684	717	Terminase small subunit

Supplementary Table 3

Sample	Replicate	Origin	Nt	Seq. depth
M1 F	1	Cromosomal	276,949	112.8
		Cromosomal	819835	92.9
		Plasmid pSR116	116032	55.9
		Plasmid pSR66	66225	36.2
		Plasmid pSR61	61538	32.0
		Plasmid pSR10	1,261	279.0
	2	Cromosomal	276,948	104.8
		Cromosomal	819835	84.9
		Plasmid pSR116	116032	54.7
		Plasmid pSR66	66225	32.8
		Plasmid pSR61	61538	29.9
		Plasmid pSR10	1,261	240.8
	3	Cromosomal	276,944	117.3
		Cromosomal	819835	95.5
		Plasmid pSR116	116032	53.7
		Plasmid pSR66	66225	36.3
		Plasmid pSR61	61538	33.3
		Plasmid pSR10	1,261	270.0
M1 EM1 F	1	Cromosomal	3528100	104.3
		Plasmid pSR116	116032	113.1
		Plasmid pSR66	66225	85.2
		Plasmid pSR61	61538	95.4
		Plasmid pSR10	1,261	337.9
		Virus EM1	35574	154.1
	2	Cromosomal	3528100	121.1
		Plasmid pSR116	116032	107.9
		Plasmid pSR66	66225	83.9
		Plasmid pSR61	61538	93.1
		Plasmid pSR10	1,261	355.7
		Virus EM1	35574	133.6
	3	Cromosomal	3528104	122.1
		Plasmid pSR116	116032	139.2
		Plasmid pSR66	66225	102.9
		Plasmid pSR61	61538	113.8
		Plasmid pSR10	1,261	395.1
		Virus EM1	35574	151.3

Supplementary Table 4

Sal. ruber strain / Virus	Primer / Probe	Sequence
Sal. ruber M1	Forward	TTCGGCCTGCCTTACTCTTT
	Reverse	TTTACCGTCCCCAACCAAGT
	Taqman probe	AGCGGAACTGCAAAGACAAGGACATGAGT
EM1 virus	Forward	GGTCGCGGGGCTTAATATC
	Reverse	CGTGTTGTTCGTTCCCCTTT
	Taqman probe	ACTGCCAACCCCGACGACTCCAC

Supplementary Table 5

Nº of cycles	Temperature	Time
1 cycle	95°C	2 minutes
30 cycles	95°C	15 seconds
	59ºC	15 seconds
	72ºC	45 seconds
1 cycle	72ºC	10 minutes
1 cycle	4ºC	∞

Supplementary Table 6

Nº of cycles	Temperature	Time
1 cycle	95°C	20 seconds
40 cycles	95°C	1 second
	60ºC	20 seconds
1 cycle	4ºC	∞

Supplementary Table 7

Region	Primer / Probe	Sequence
Sodium/glucose cotransporter	205F	GGGGAAGGAGCAGCTTCAG
	414R	AGCTGATGTACCGCAACGAC

Supplementary Table 8

Nº of cycles	Temperature	Time
1 cycle	95°C	2 minutes
30 cycles	95°C	15 seconds
	59ºC	15 seconds
	72ºC	1 min 15 seconds
1 cycle	72ºC	10 minutes
1 cycle	4ºC	∞

Supplementary Table 9

	PFU/ml				
**Time (h)**	**Replicate 1**	**Replicate 2**	**Replicate 3**	**Mean**	**SD**
0	140	150	140	1.43E+02	5.77E+00
17	180	200	150	1.77E+02	2.52E+01
19	250	270	230	2.50E+02	2.00E+01
21	280	250	290	2.73E+02	2.08E+01
23	450	780	620	6.17E+02	1.65E+02
25	1050	1160	1000	1.07E+03	8.19E+01
27	1860	1950	1720	1.84E+03	1.16E+02
29	1940	1900	2010	1.95E+03	5.57E+01
31	1990	2140	1940	2.02E+03	1.04E+02

Free PFU/ml after initial adsorption				
**Replicate 1**	**Replicate 2**	**Replicate 3**	**Mean**	**SD**
40	30	20	30	10

Burst size
11
